# Supplementary material for: Protein thermal sensing regulates physiological amyloid aggregation
Source: Nat Commun. 2024 Feb 9;15:1222. doi: 10.1038/s41467-024-45536-0 (PMC10858206; doi:10.1038/s41467-024-45536-0)
Supplement: Supplementary file 1 — Supplementary Information [file 41467_2024_45536_MOESM1_ESM.pdf]

Supplementary Material For

Protein Thermal Sensing Regulates Physiological Amyloid Aggregation

Dane Marijan[1,2], Evgenia A. Momchilova[1,2,†], Daniel Burns[3,†], Sahil Chandhok[1,2,†], Richard Zapf[1,2], Holger Wille[4,5,6], Davit A Potoyan[3,7], Timothy E. Audas[1,2*]

[1]Department of Molecular Biology and Biochemistry, Simon Fraser University, 8888 University Drive, Burnaby, BC V5A 1S6, Canada
[2]Centre for Cell Biology, Development, and Disease, Simon Fraser University, 8888 University Drive, Burnaby, BC V5A 1S6, Canada
[3]Roy J. Carver Department of Biochemistry, Biophysics and Molecular Biology, Iowa State University, Ames, IA 50011, USA
[4]Department of Biochemistry, University of Alberta, Edmonton, Alberta, T6G 2H7, Canada
[5]Centre for Prions and Protein Folding Diseases, University of Alberta, Edmonton, Alberta, T6G 2M8, Canada
[6]Neuroscience and Mental Health Institute, University of Alberta, Edmonton, Alberta, T6G 2E1, Canada
[7]Department of Chemistry, Iowa State University, Ames, IA 50011, USA
† These authors contributed equally to this work
*Corresponding author: taudas@sfu.ca

The PDF file includes:
Supplementary Figures 1-7
Supplementary Table 1
Supplementary Figure and Table legends

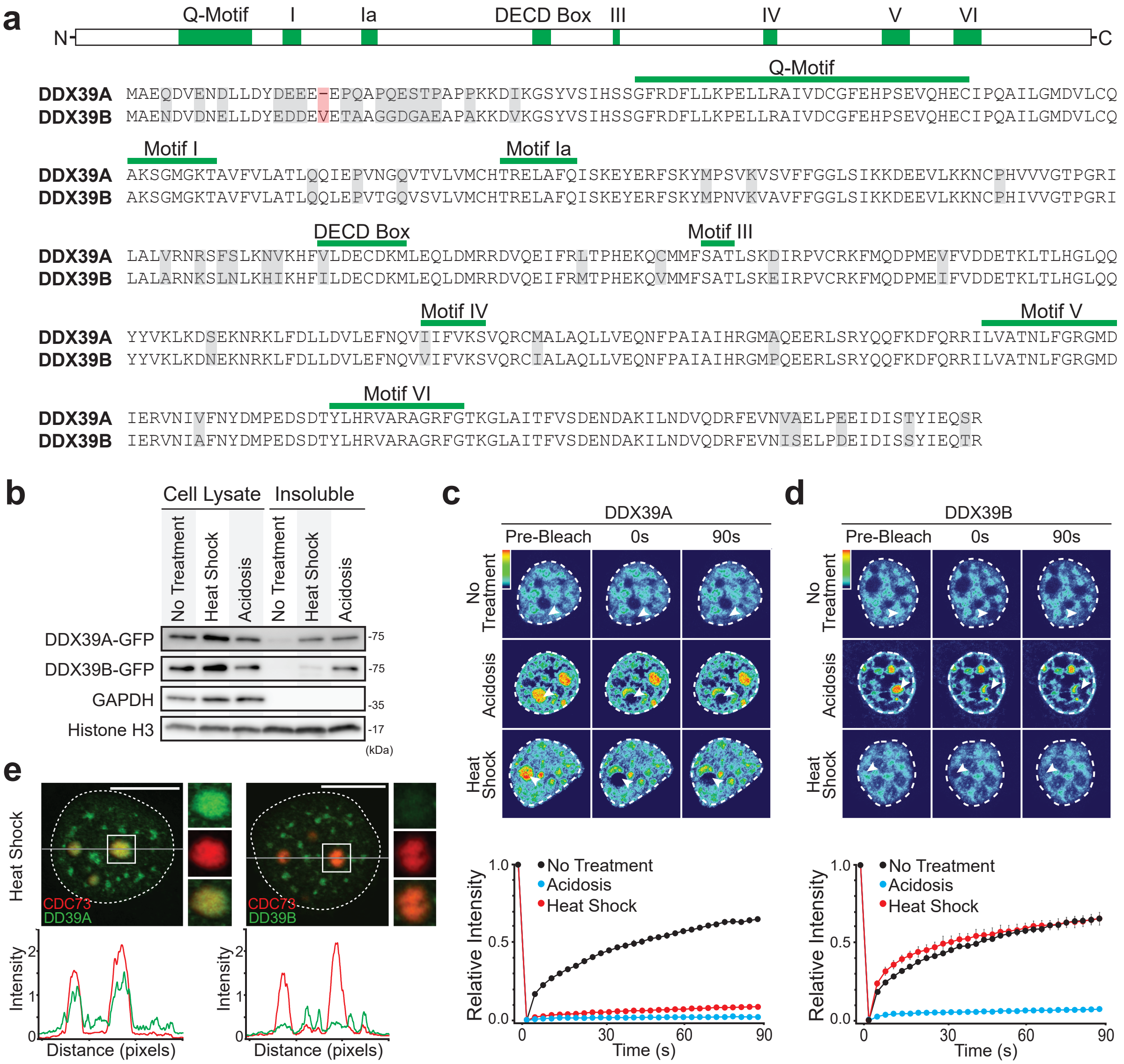

Supplementary Fig. 1: DDX39A and DDX39B are differentially targeted, immobilized, and insoluble in A-bodies.

a Schematic of the DDX39 proteins with the locations of hallmark RNA helicase domains indicated (top). The complete protein sequences of DDX39A and DDX39B were aligned (bottom), and divergent amino acid residues are highlighted (grey). DDX39B contains an additional amino acid (red), leading to the positions of critical thermo-sensing residues being offset by 1. **b** MCF-7 cells expressing DDX39A-GFP or DDX39B-GFP were untreated, heat shocked or exposed to acidotic conditions. Whole cell lysates and insoluble fractions were extracted and assayed by western blotting. GAPDH (soluble) and Histone H3 (insoluble) were used as fractionation controls. **c-d** MCF-7 cells expressing DDX39A (c) and DDX39B (d) were bleached in the indicated region (arrowhead) and allowed to recover. Representative pre-bleach, post-bleach (0s), and recovery (90s) images are presented in pseudo-color with high (red) to low (blue) intensity indicating changes in fluorescence (upper panels). Fluorescence recovery kinetics of the bleached regions were quantified and presented (lower panels) as the mean relative intensity of 10 cells per replicate. Error bars represent ± s.e.m (n = 3 independent experiments). **e** DDX39A-GFP or DDX39B-GFP were co-expressed in heat shock treated MCF-7 cells with the A-body marker molecule CDC73-mCherry. Green and red fluorescence signal intensity were calculated across the nuclei (grey line) and blotted below the microscopy images.

Dashed circles in microscopy images represent nuclei, selected regions (white boxes) are expanded to the right (merge: bottom), and white scale bars represent 10 μm. Source data for all graphs and blots are provided with this paper.

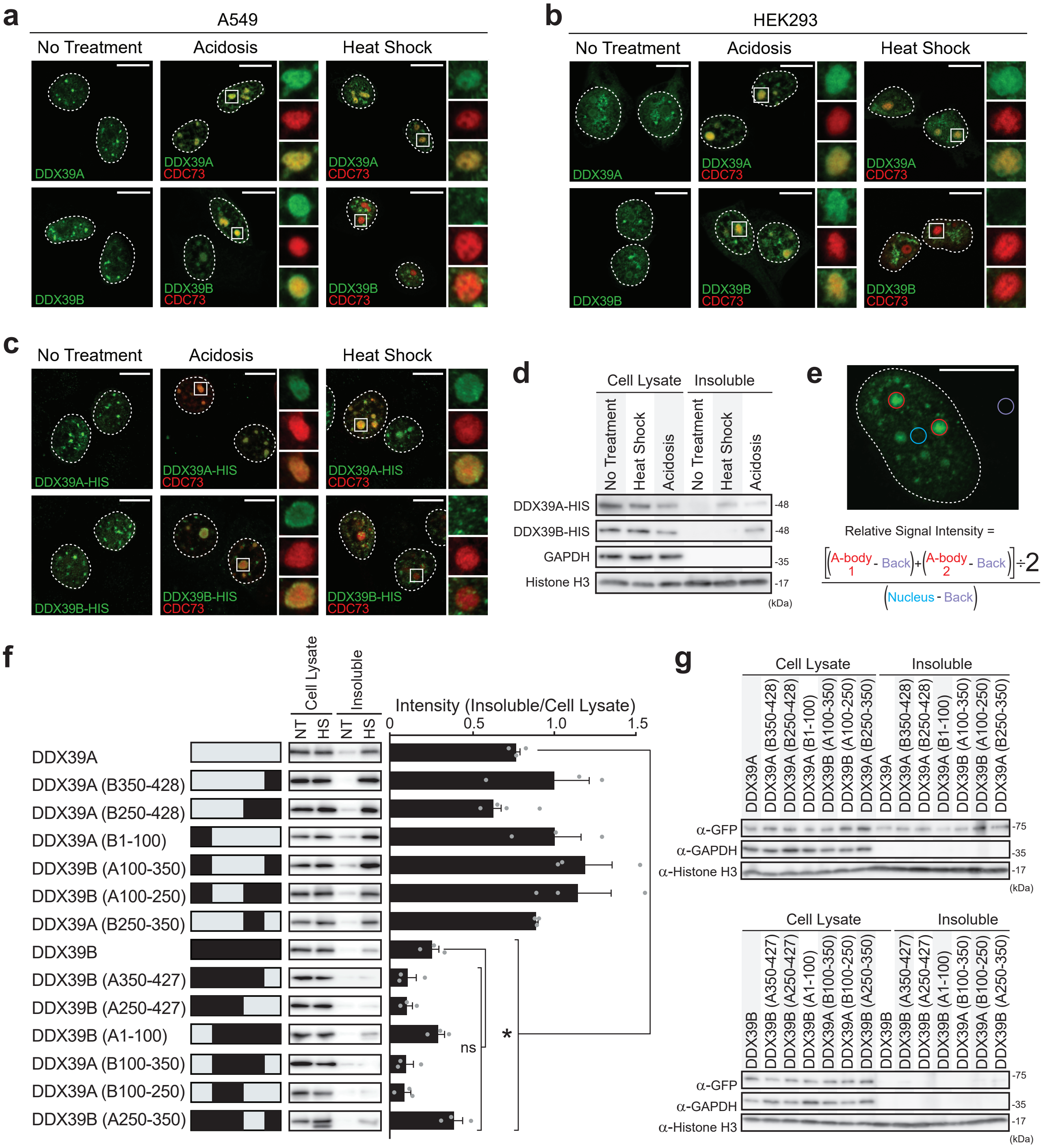

a
A549
No Treatment
Acidosis
Heat Shock
DDX39A
DDX39A CDC73
DDX39B
DDX39B CDC73
b
HEK293
No Treatment
Acidosis
Heat Shock
DDX39A
DDX39A CDC73
DDX39B
DDX39B CDC73
c
No Treatment
Acidosis
Heat Shock
DDX39A-HIS
DDX39A-HIS CDC73
DDX39B-HIS
DDX39B-HIS CDC73
d
Cell Lysate
Insoluble
No Treatment
Heat Shock
Acidosis
DDX39A-HIS
DDX39B-HIS
GAPDH
Histone H3
-48
-35
-17
(kDa)
e
Relative Signal Intensity =
[(A-body 1 - Back)+(A-body 2 - Back)]÷2
(Nucleus - Back)
f
Cell Lysate
Insoluble
NT HS NT HS
Intensity (Insoluble/Cell Lysate)
0 0.5 1.0 1.5
DDX39A
DDX39A (B350-428)
DDX39A (B250-428)
DDX39A (B1-100)
DDX39B (A100-350)
DDX39B (A100-250)
DDX39A (B250-350)
DDX39B
DDX39B (A350-427)
DDX39B (A250-427)
DDX39B (A1-100)
DDX39A (B100-350)
DDX39A (B100-250)
DDX39B (A250-350)
ns
*
g
Cell Lysate
Insoluble
DDX39A
DDX39A (B350-428)
DDX39A (B250-428)
DDX39A (B1-100)
DDX39B (A100-350)
DDX39B (A100-250)
DDX39A (B250-350)
α-GFP
α-GAPDH
α-Histone H3
-75
-35
-17
(kDa)
Cell Lysate
Insoluble
DDX39B
DDX39B (A350-427)
DDX39B (A250-427)
DDX39B (A1-100)
DDX39A (B100-350)
DDX39A (B100-250)
DDX39B (A250-350)
α-GFP
α-GAPDH
α-Histone H3
-75
-35
-17
(kDa)

Supplementary Fig. 2: Heat shock-specific aggregation is conserved across multiple cell lines and regulated by a central region of DDX39A and DDX39B.

a A549 and **b** HEK293 cells expressing DDX39A-GFP or DDX39B-GFP and the A-body marker protein CDC73-mCherry were left untreated or exposed to heat shock or acidotic conditions. **c** MCF-7 cells expressing the A-body marker CDC73-mCherry and the his-tagged proteins DDX39A-8xHis or DDX39B-8xHis were treated as in (a), prior to immunostaining with an -His antibody and imaging. **d** DDX39A-8xHis or DDX39B-8xHis were detected in fractionated MCF-7 cells subjected to the indicated conditions by western blot. GAPDH and Histone H3 were used as fractionation controls. **e** Relative A-body Intensity was calculated by subtracting the average intensity of two A-bodies (red circles) from the background signal (lavender circle) and dividing this by the background subtracted nuclear intensity (blue circle). Sample cell (top) and equation (bottom) are presented. **f** A range of DDX39 substitution constructs (left panel) were expressed in untreated (NT) or heat shocked (HS) cells prior to cell lysate extraction and insoluble fractionation. Representative western blots are included (central panel) and quantified (right panel). Bars represent the average band intensity ratio of the insoluble (heat shock) fraction relative to whole cell lysate. Error bars represent ± s.e.m (n = 3 independent experiments, a two-tailed Student's t-test was used: $*p \leq 0.01$, not significant: ns). **g** Additional, representative western blots of the samples used in (f). Soluble (GAPDH) and insoluble (Histone H3) fractionation controls are included.

Dashed circles in microscopy images represent nuclei, and selected regions (white boxes) were expanded to the right (merge: bottom). White scale bars represent 10 μm. Source data for all graphs and blots are provided with this paper.

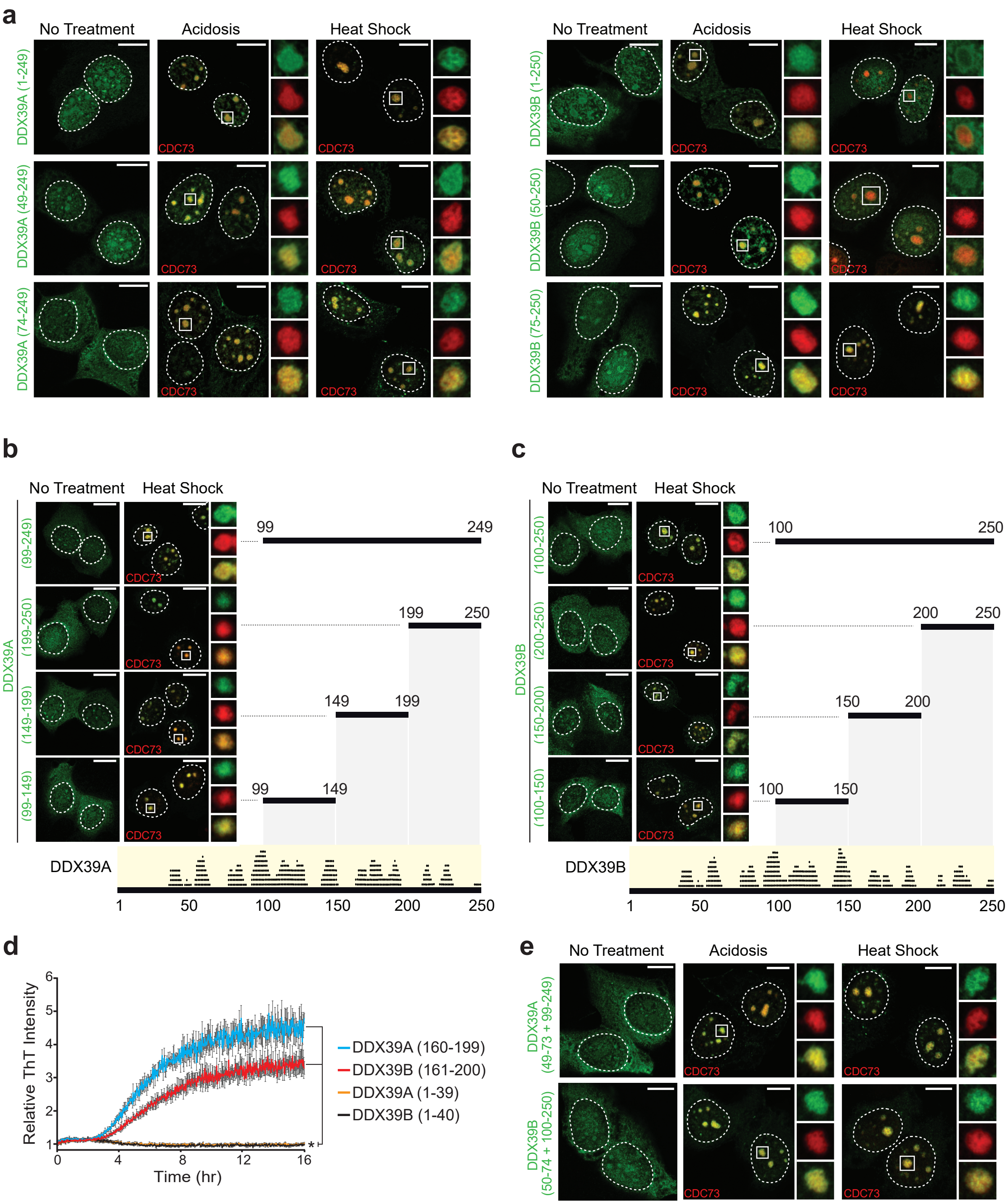

a
No Treatment
Acidosis
Heat Shock
DDX39A (1-249)
DDX39A (49-249)
DDX39A (74-249)
DDX39B (1-250)
DDX39B (50-250)
DDX39B (75-250)
CDC73
b
DDX39A
(99-249)
(199-250)
(149-199)
(99-149)
c
DDX39B
(100-250)
(200-250)
(150-200)
(100-150)
d
Relative ThT Intensity
Time (hr)
DDX39A (160-199)
DDX39B (161-200)
DDX39A (1-39)
DDX39B (1-40)
e
DDX39A (49-73 + 99-249)
DDX39B (50-74 + 100-250)

Supplementary Fig. 3: Identification of minimal aggregation prone A-body targeting motif(s) in DDX39A and DDX39B.

a MCF-7 cells were transfected with plasmids encoding DDX39A (1-249)-GFP, DDX39A (49-249)-GFP, DDX39A (74-249)-GFP, DDX39B (1-250)-GFP, DDX39B (50-250)-GFP, or DDX39B (75-250)-GFP, and the A-body marker CDC73-mCherry. Samples were left untreated or exposed to heat shock or acidosis treatment prior to fixation and visualization. **b-c** MCF-7 cells expressing the indicated DDX39A-GFP (b) or DDX39B-GFP (c) construct and CDC73-mCherry were left untreated or exposed to heat shock conditions. Schematic diagrams of the constructs (right) are aligned with representative images (left) and mapped to the consensus aggregation propensity of the N-terminal region of DDX39B or DDX39B (bottom). Individual lines/dots represent amino acid positions predicted to be within aggregation prone regions, vertically stacked dots indicate prediction by multiple algorithms via AmylPred2. **d** A Thioflavin T assay was used to measure in vitro aggregation kinetics for the DDX39A (1-39), DDX39A (160-199), DDX39B (1-40), and DDX39B (161-200) peptides over a 16-hr time frame. Mean fluorescent intensity is relative to the no peptide sample. Error bars represent ± s.e.m (n = 3 independent experiments, a two-tailed Student's t-test was used: $^*p \leq 0.05$). **e** DDX39A (49-73+99-249)-GFP or DDX39B (50-74+100-250)-GFP and CDC73-mCherry were co-expressed in MCF-7 cells left untreated or exposed to heat shock or acidotic conditions. Representative images are presented.

Dashed circles in microscopy images represent nuclei, and selected regions (white boxes) were expanded to the right (merge: bottom). White scale bars represent 10 μm. Source data for graphs are provided with this paper.

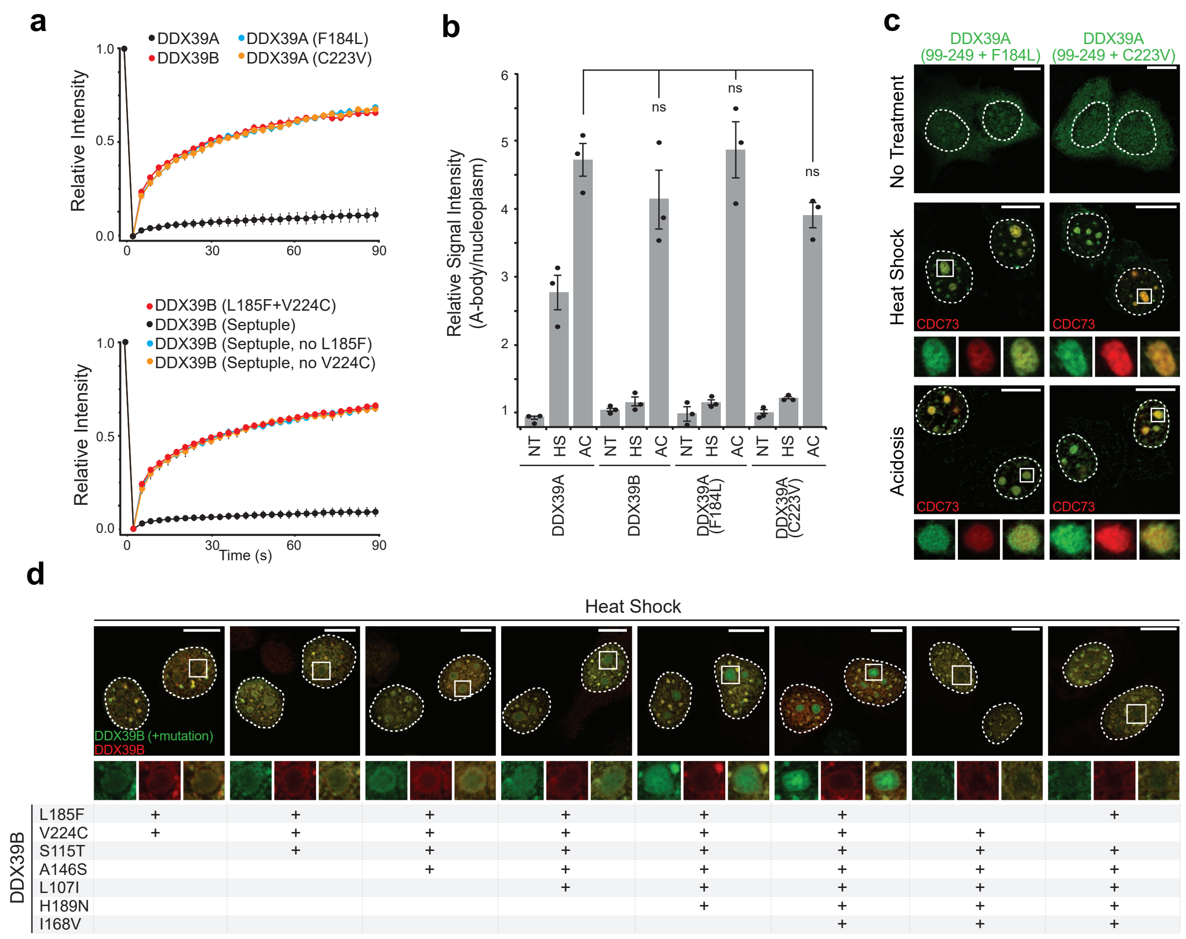

Supplementary Fig. 4: Central residues control DDX39A and DDX39B heat shock-specific A-body targeting.

a Quantification of FRAP results for heat shock-treated (4h) MCF-7 cells expressing DDX39A-GFP, DDX39B-GFP, DDX39A (F184L)-GFP, DDX39A (C223V)-GFP, DDX39B (Septuplet: L185F+V224C+S115T+A146S+L107I+H189N+I168V), DDX39B (Septuplet, no L185F: V224C+S115T+A146S+L107I+H189N+I168V), or DDX39B (Septuplet, no V224C: L185F+S115T+A146S+L107I+H189N+I168V). Fluorescence recovery kinetics of the bleached regions are presented as the mean relative intensity of 10 cells per replicate. Error bars represent ± s.e.m. (n=3 independent experiments). **b** MCF-7 cells expressing DDX39A-GFP, DDX39B-GFP, DDX39A (F184L)-GFP, or DDX39A (C223V)-GFP were left untreated or exposed to heat shock or acidosis treatment prior to imaging and relative A-body intensity quantification. For each sample 10 cells were analyzed per replicate, and values represent means ± s.e.m (n = 3 independent experiments, a two-tailed Student's t-test was used: *p ≤ 0.05). **c** MCF-7 cells expressing DDX39A (99-249+F184L)-GFP or DDX39A (99-249+C223V)-GFP and the A-body marker CDC73-mCherry were left untreated, heat shocked or exposed to acidotic conditions prior to visualization. **d** Heat shock-treated MCF-7 cells expressing DDX39B-GFP with the indicated amino acid substitutions (table below) were visualized for A-body recruitment. Wild-type DDX39B-mCherry was co-expressed as a control.

Dashed circles in microscopy images represent nuclei, selected regions (white boxes) were expanded below (merge: far right). White scale bars represent 10 μm. Source data for graphs are provided with this paper.

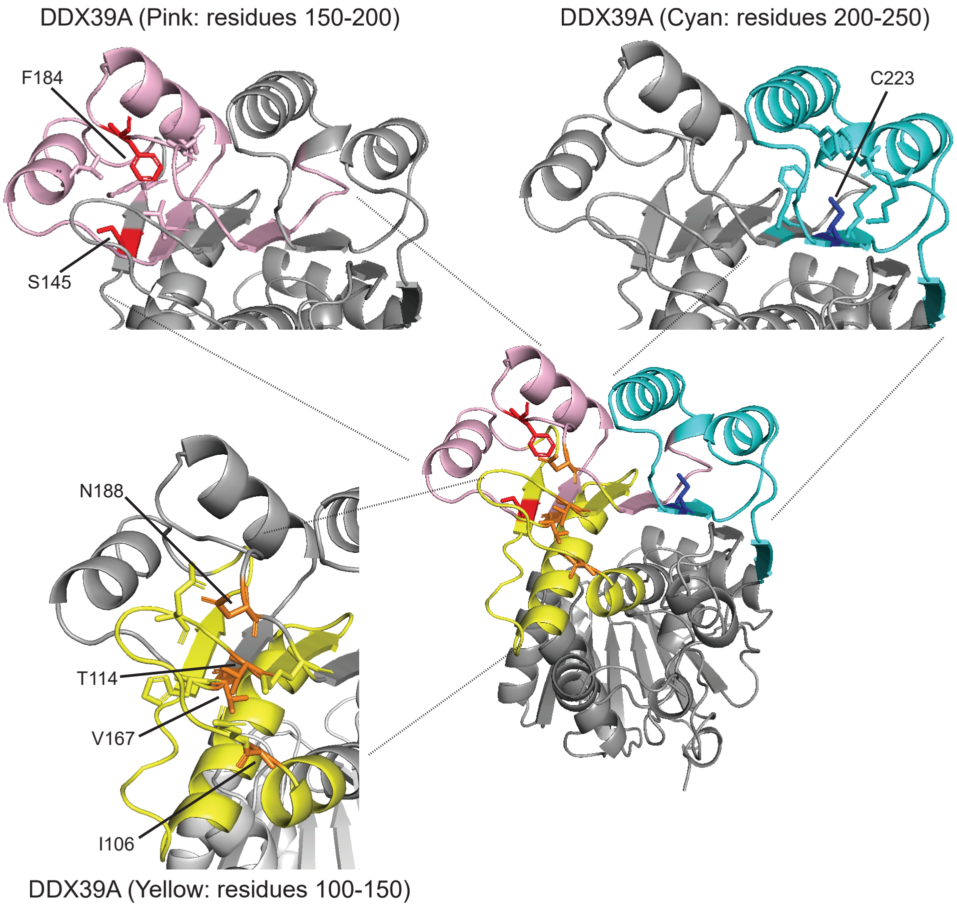

Supplementary Fig. 5: Heat shock regulatory pockets align with the minimal A-body targeting motifs.

Cartoon of the predicted DDX39A 3D structure with the minimal A-body targeting motifs highlighted in yellow (100-150), pink (150-200), and cyan (200-250). Enlarged structures highlight the individual minimal A-body targeting motifs, and the critical structural residues F184 and S145 (red), C223 (blue), and T114, I106, N188 and V167 (orange). The residues found in the surrounding regulatory pockets are presented as yellow, pink, and cyan sticks, respectively.

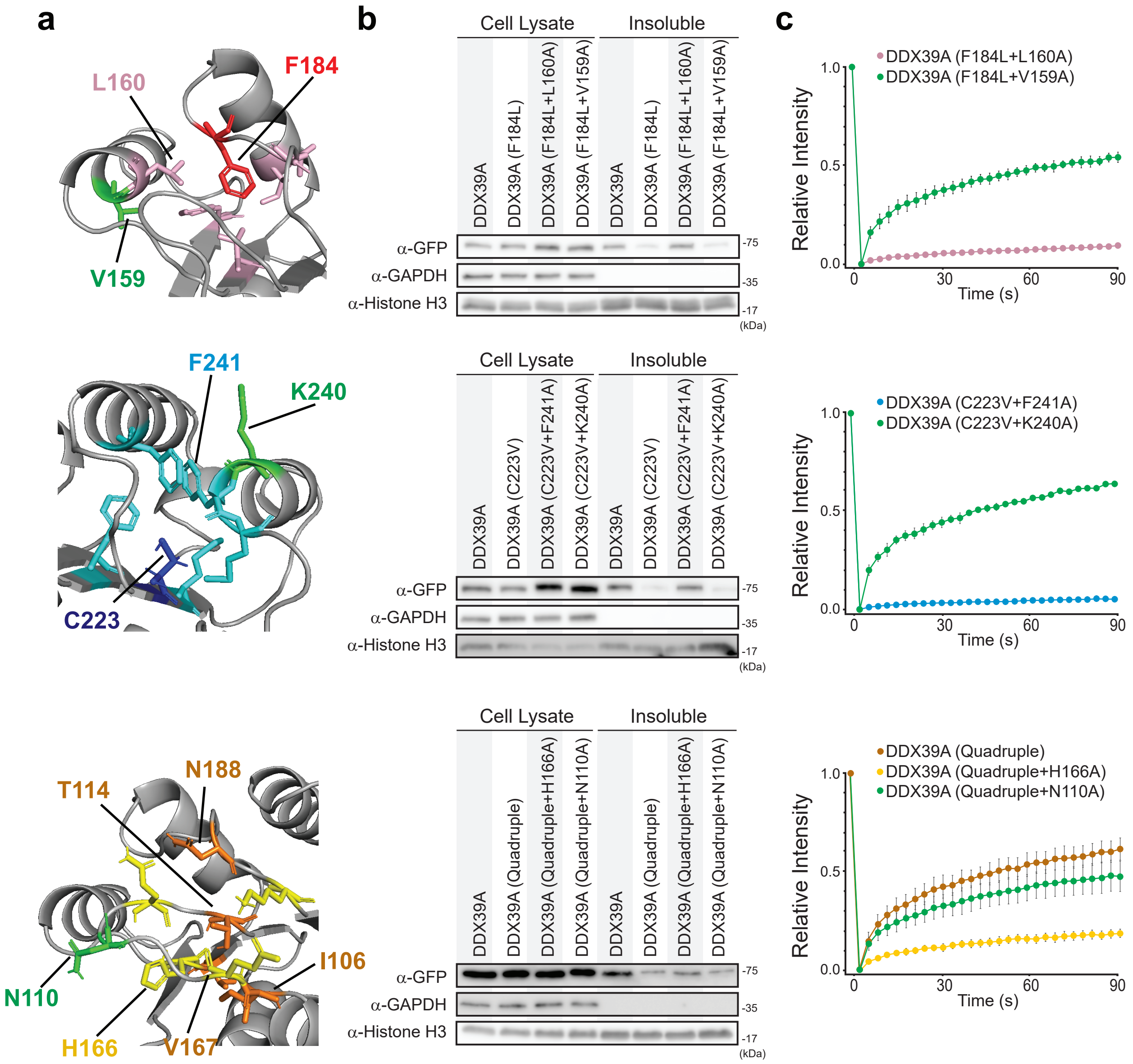

Supplementary Fig. 6: Mutations in the regulatory pockets restore A-body targeting of DDX39A.

a Cartoon DDX39A structures, with the pockets surrounding F184 (red, top), C223 (blue, middle) and Quadruple (T114, I106, N188, V167: orange, bottom) indicated. Structural pocket residues substituted in panels (b-c) are denoted pink (top), cyan (middle) and yellow (bottom), while the control residues outside of pockets are indicated in green. **b** Heat shock-treated MCF-7 cells expressing the indicated DDX39A-GFP substitutions were lysed, with whole cell lysates and insoluble fractions extracted. Western blotting to detect the DDX39A protein (α-GFP), GAPDH (soluble) and Histone H3 (insoluble) was performed. **c** Quantification of FRAP results for heat shock-treated (4h) MCF-7 cells expressing DDX39A (F184L+L160A), DDX39A (F184L+V159A), DDX39A (C223V+F241A), DDX39A (C223V+K240A), DDX39A (Quadruple: T114S+I106L+N188H+V167I), DDX39A (Quadruple+H166A: T114S+I106L+N188H+V167I+H166A), or DDX39A (Quadruple +N110A: T114S+I106L+N188H+V167I+N110A). Fluorescence recovery kinetics are presented as the mean relative intensity of 10 cells per replicate. Error bars represent ± s.e.m. (n = 3 independent experiments). Source data for all graphs and blots are provided with this paper.

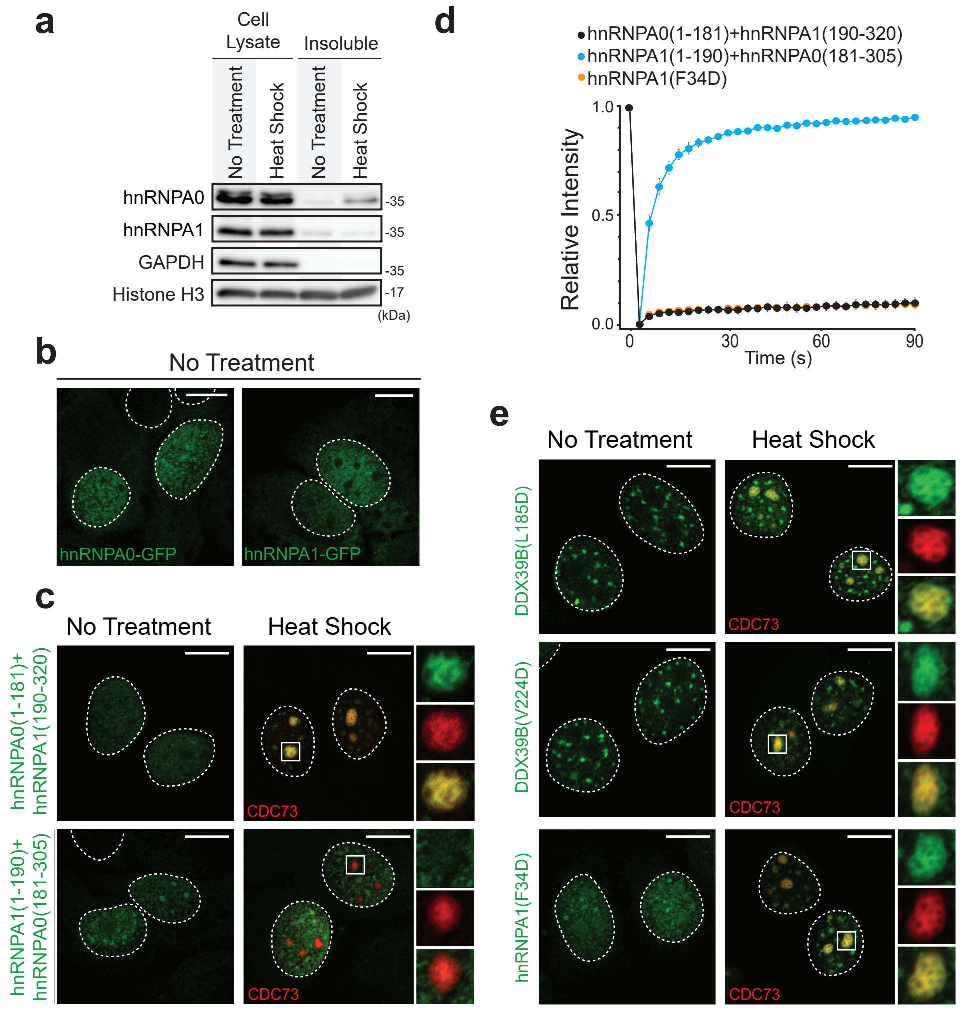

Supplementary Fig. 7: Analogous structural pockets regulate DDX39B and hnRNPA1 A-body targeting.

a Untreated or heat shocked MCF-7 cells were lysed, with whole cell lysates and insoluble fractions extracted. Western blotting to detect endogenously expressed hnRNPA0 (α-hnRNPA0), hnRNPA1 (α-hnRNPA1), GAPDH (soluble) and Histone H3 (insoluble) was performed. **b** Untreated MCF-7 cells expressing hnRNPA0-GFP or hnRNPA1-GFP were imaged. **c** MCF-7 cells expressing the A-body marker CDC73-mCherry and the indicated hnRNPA-GFP constructs were untreated or heat shocked and imaged. **d** Quantification of FRAP results for heat shock-treated (4h) MCF-7 cells expressing indicated hnRNPA proteins. Fluorescence recovery kinetics are presented as the mean relative intensity of 10 cells per replicate. Error bars represent ± s.e.m. (n = 3 independent experiments). **e** MCF-7 cells expressing the A-body marker CDC73-mCherry and DDX39B (L185D)-GFP, DDX39B (V224D)-GFP, or hnRNPA1 (F34D)-GFP were untreated or heat shocked and imaged.

Dashed circles in microscopy images represent nuclei, selected regions (white boxes) are expanded (merge: bottom), white scale bars represent 10 μm. Source data for all graphs and blots are provided with this paper.

Simulation Protein	Total Number of Water Molecules	Total Number of Atoms	Box Dimensions	Salt Concentration
**DDX39A**	29,660	95,296	100 A3	0.15 M
**DDX39B**	29,239	94,045	99 A3	0.15 M

Supplementary Table 1: Setup for the molecular dynamics simulations of DDX39A and DDX39B.
