# Supplementary material for: Protein thermal sensing regulates physiological amyloid aggregation
Source: Nat Commun. 2024 Feb 9;15:1222. doi: 10.1038/s41467-024-45536-0 (PMC10858206; doi:10.1038/s41467-024-45536-0)
Supplement: Supplementary file 3 — Reporting Summary [file 41467_2024_45536_MOESM3_ESM.pdf]

Corresponding author(s): Timothy Audas

Last updated by author(s): Jan 16, 2024

Reporting Summary

Nature Portfolio wishes to improve the reproducibility of the work that we publish. This form provides structure for consistency and transparency in reporting. For further information on Nature Portfolio policies, see our Editorial Policies and the Editorial Policy Checklist.

Statistics

For all statistical analyses, confirm that the following items are present in the figure legend, table legend, main text, or Methods section.

n/a	Confirmed	
☐	☒	The exact sample size (*n*) for each experimental group/condition, given as a discrete number and unit of measurement
☐	☒	A statement on whether measurements were taken from distinct samples or whether the same sample was measured repeatedly
☐	☒	The statistical test(s) used AND whether they are one- or two-sided<br>*Only common tests should be described solely by name; describe more complex techniques in the Methods section.*
☒	☐	A description of all covariates tested
☒	☐	A description of any assumptions or corrections, such as tests of normality and adjustment for multiple comparisons
☐	☒	A full description of the statistical parameters including central tendency (e.g. means) or other basic estimates (e.g. regression coefficient) AND variation (e.g. standard deviation) or associated estimates of uncertainty (e.g. confidence intervals)
☐	☒	For null hypothesis testing, the test statistic (e.g. *F*, *t*, *r*) with confidence intervals, effect sizes, degrees of freedom and *P* value noted<br>*Give P values as exact values whenever suitable.*
☒	☐	For Bayesian analysis, information on the choice of priors and Markov chain Monte Carlo settings
☒	☐	For hierarchical and complex designs, identification of the appropriate level for tests and full reporting of outcomes
☒	☐	Estimates of effect sizes (e.g. Cohen's *d*, Pearson's *r*), indicating how they were calculated

Our web collection on statistics for biologists contains articles on many of the points above.

Software and code

Policy information about availability of computer code

Data collection	Fluorescent Imaging was performed on a Carl Zeiss LSM880 confocal laser scanning microscope with Airyscan using Zen 2.3 (black edition)<br>Thioflavin fibrillation data was collected using the Tecan Infinite M200 Pro microplate reader<br>Immunoblot images were scanned on the Amerhsam Imager 600 (GE Healthcare Life Sciences)<br>Simulations are performed with molecular dynamics library OpenMM 7.6
Data analysis	Image processing was performed using Carl Zeiss Zen 3.1 (blue edition), no non-linear adjustments were made<br>Fluorescence Recovery After Photobleaching and western intensity analyses were done on Image J 1.52a (National Institute of Health)<br>Microscopy and Western blot cropping was performed in Adobe Photoshop 2021<br>Disordered protein regions were predicted using the IUPred 3 interface (https://iupred3.elte.hu/)<br>Consensus aggregation propensity was calculated using AmylPred 2 (http://thalis.biol.uoa.gr/AMYLPRED2/)<br>MDAnalysis python library calculated the RMSF and RMSD of the C alpha atoms<br>Structure models were generated, analyzed, and presented using version 2 of the PyMOL Molecular Graphics System (Schrödinger, LLC.)<br>Protein structural predictions were performed using the Fold and Function Assignment System (https://bio.tools/ffas)<br>Microsoft Excel 2016 was used to perform basic statistical analysis<br>Graphs were prepared in Microsoft Excel 2016 and GraphPad Prism 10

For manuscripts utilizing custom algorithms or software that are central to the research but not yet described in published literature, software must be made available to editors and reviewers. We strongly encourage code deposition in a community repository (e.g. GitHub). See the Nature Portfolio guidelines for submitting code & software for further information.

Data

Policy information about availability of data

All manuscripts must include a data availability statement. This statement should provide the following information, where applicable:

- Accession codes, unique identifiers, or web links for publicly available datasets
- A description of any restrictions on data availability
- For clinical datasets or third party data, please ensure that the statement adheres to our policy

The paper and supplementary information contains all the data needed to evaluate the conclusions of the work. Raw data and p values for all Figures and Supplementary Figures are present in the Source Data files. Initial coordinate, simulation input files, and coordinate files of the final output of the molecular dynamics simulations are available online (https://github.com/PotoyanGroup). DDX39B (AF-Q13838-F1 and PDB: 1XTI), hnRNPA0 (AF-Q13151-F1), hnRNPA1 (AF-P09651-F1 and PDB: 1L3K) protein structures were used in this work. Any additional information is available upon request to the corresponding author (T.E.A: taudas@sfu.ca).

Research involving human participants, their data, or biological material

Policy information about studies with human participants or human data. See also policy information about sex, gender (identity/presentation), and sexual orientation and race, ethnicity and racism.

Reporting on sex and gender	Not Applicable
Reporting on race, ethnicity, or other socially relevant groupings	Not Applicable
Population characteristics	Not Applicable
Recruitment	Not Applicable
Ethics oversight	Not Applicable

Note that full information on the approval of the study protocol must also be provided in the manuscript.

Field-specific reporting

Please select the one below that is the best fit for your research. If you are not sure, read the appropriate sections before making your selection.

☒ Life sciences ☐ Behavioural & social sciences ☐ Ecological, evolutionary & environmental sciences

For a reference copy of the document with all sections, see nature.com/documents/nr-reporting-summary-flat.pdf

Life sciences study design

All studies must disclose on these points even when the disclosure is negative.

Sample size	No sample size calculation was applied in this study to predetermine sample sizes for experiments using cell lines. A sample size of three was used and was determined based upon other studies with similar methodologies (PMID: 37968398 , 27720612, 27708256). The sample number analyzed is indicated in the figure legends and Methods section.
Data exclusions	No data were excluded from the analysis.
Replication	Each experiment was repeated independently at least three times, which means that the experiments were done on different days, withcells from different passages. All attempts at replication were successful.
Randomization	Samples/organisms/participants were not allocated into groups, experimental variables were selected and manipulated to generate samples. Thus, randomization was not necessary in this study.
Blinding	Based on the mode of data generation (above), it was not possible to blind the investigator to the groups/samples.

Reporting for specific materials, systems and methods

We require information from authors about some types of materials, experimental systems and methods used in many studies. Here, indicate whether each material, system or method listed is relevant to your study. If you are not sure if a list item applies to your research, read the appropriate section before selecting a response.

Materials & experimental systems

n/a	Involved in the study	
☐	☒	Antibodies
☐	☒	Eukaryotic cell lines
☒	☐	Palaeontology and archaeology
☒	☐	Animals and other organisms
☒	☐	Clinical data
☒	☐	Dual use research of concern
☒	☐	Plants

Methods

n/a	Involved in the study	
☒	☐	ChIP-seq
☒	☐	Flow cytometry
☒	☐	MRI-based neuroimaging

Antibodies

Antibodies used	IHC Primary antibody: hnRNPA1 - ThermoFisher Scientific, PA5-19431, polyclonal, 1:200<br>IHC Primary antibody: hnRNPA0 - ThermoFisher Scientific, PA5-57722, polyclonal, 1:200<br>IHC Primary antibody: His antibody - ThermoFisher Scientific, MA1-21315, monoclonal (HIS.H8), 1:1000<br>IHC Secondary antibody: anti-rabbit Alexa Fluor 488 - ThermoFisher Scientific, A11008, polyclonal, 1:400<br>IHC Secondary antibody: anti-mouse Alexa Fluor 488 - ThermoFisher Scientific, A11001, polyclonal, 1:400<br>Western Blot Primary antibody: GFP - Santa Cruz Biotechnology, sc-9996, monoclonal (B-2), 1:1,000<br>Western Blot Primary antibody: GAPDH - Santa Cruz Biotechnology, sc-47724, monoclonal (0411), 1:4,000<br>Western Blot Primary antibody: Histone H3 - Cell Signaling Technology, 9715, polyclonal, 1:10,000<br>Western Blot Primary antibody: hnRNPA1 - ThermoFisher Scientific, PA5-19431, polyclonal, 1:1,000<br>Western Blot Primary antibody: hnRNPA0 - ThermoFisher Scientific, PA5-57722, polyclonal, 1:1,000<br>Western Blot Primary antibody: His antibody - ThermoFisher Scientific, MA1-21315, monoclonal (HIS.H8), 1:1000<br>Western Blot Secondary antibody: anti-rabbit HRP - ThermoFisher Scientific, A16023, polyclonal, 1:10,000<br>Western Blot Secondary antibody: anti-mouse HRP - ThermoFisher Scientific, A16011, polyclonal, 1:10,000
Validation	hnRNPA1 - ThermoFisher Scientific, PA5-19431: Antibody have undergone advanced verification by manufacturer (https://www.thermofisher.com/antibody/product/hnRNP-A1-Antibody-Polyclonal/PA5-19431)<br>hnRNPA0 - ThermoFisher Scientific, PA5-57722: Antibody have undergone advanced verification by manufacturer (https://www.thermofisher.com/antibody/product/HNRNPA0-Antibody-Polyclonal/PA5-57722)<br>His antibody - ThermoFisher Scientific, MA1-21315: 1113 citations reported on manufacturer's website (https://www.cellsignal.com/products/primary-antibodies/histone-h3-antibody/9715)<br>GFP - Santa Cruz Biotechnology, sc-9996: 3296 citations reported on manufacturer's website (https://www.scbt.com/p/gfp-antibody-b-2)<br>GAPDH - Santa Cruz Biotechnology, sc-47724: 3113 citations reported on manufacturer's website (https://www.scbt.com/p/gapdh-antibody-0411)<br>Histone H3 - Cell Signaling Technology, 9715: 1113 citations reported on manufacturer's website (https://www.cellsignal.com/products/primary-antibodies/histone-h3-antibody/9715)

Eukaryotic cell lines

Policy information about cell lines and Sex and Gender in Research

Cell line source(s)	MCF-7, A549, and HEK293 cells were purchased from ATCC (Manassas, VA, USA)
Authentication	Applied Biological Material (ABM) human cell line authentication was done for the MCF-7 cells. The report indicated that these cells share 20/20 alleles; 100% identity (CLASTR 1.4.4 STR Similarity Search Tool score) with the profile of the commercial MCF-7 line (CVCL_0031), whose profile can be found online (http://web.expasy.org/cellosaurus/CVCL_0031), and that these cells can be considered to be derived from a common ancestor.<br>A549 and HEK293 were authenticated using morphology analyses and were carefully labeled and tracked.
Mycoplasma contamination	Applied Biological Material (ABM) Mycoplasma PCR Detection Kit (Cat# G238) confirmed that cells used in this study wer mycoplasm negative.
Commonly misidentified lines<br>(See ICLAC register)	No commonly misidentified cell lines were used in this study.

Plants

Seed stocks	Not Applicable
Novel plant genotypes	Not Applicable
Authentication	Not Applicable
